# Complete Genome Sequence of Carbapenem-Resistant *Klebsiella pneumoniae* Strain 1756, Isolated from a Pus Specimen

**DOI:** 10.1128/genomeA.00066-17

**Published:** 2017-03-30

**Authors:** Cheng-Yen Kao, Jing-Jou Yan, Yu-Chun Lin, Po-Xing Zheng, Jiunn-Jong Wu

**Affiliations:** a Department of Biotechnology and Laboratory Science in Medicine, School of Biomedical Science and Engineering, National Yang Ming University, Taipei, Taiwan; b Department of Pathology, College of Medicine, National Cheng Kung University, Tainan, Taiwan; c Department of Medical Laboratory Science and Biotechnology, National Cheng Kung University, Tainan, Taiwan

## Abstract

Carbapenem-resistant *Klebsiella pneumoniae* strain 1756 was isolated from a pus specimen from a Taiwanese patient. Here, the complete genome sequence of strain 1756 is presented.

## GENOME ANNOUNCEMENT

*Klebsiella pneumoniae* is a Gram-negative opportunistic bacillus in the family *Enterobacteriaceae* with worldwide distribution that causes pneumonia, liver abscess, septicemia, and urinary tract infection in humans. Carbapenems are considered to be the most effective broad-spectrum β-lactam antimicrobial agents for the defense of multidrug resistant Gram-negative organisms, including *K. pneumoniae*. However, over the past decade the carbapenem resistances of *Enterobacteriaceae*, *Pseudomonas aeruginosa*, and *Acinetobacter baumannii* have emerged as significant public health threats (1, 2). Here, we report the complete genome sequence of *K. pneumoniae* strain 1756, isolated from a pus specimen from a 63-year-old Taiwanese women.

*K. pneumoniae* strain 1756 was sequenced using the PacBio RS II platform (Pacific Biosciences, USA), generating a library containing 76,075 single reads with an average length of 14,567 bp and a 173-fold average coverage. Reads were assembled using HGAP version 3.0, which returned three contigs. One short contig (30,002 bp) without a plasmid replicon was discarded because the assembly was problematic. For the remaining two contigs, the head segment was almost identical to the tail segment, indicating the circular nature of the contigs.

The 1756 genome has a size of 5,195,816 bp and a G+C content of 57.6%. Sequences were annotated at NCBI (http://www.ncbi.nlm.nih.gov/genome/annotation_prok) or with the RAST prokaryotic genome annotation server (http://rast.nmpdr.org/) (3). RAST annotation results showed that the genome contained 4,856 coding sequences and 111 RNA genes. The MLST, ResFinder, and PlasmidFinder (http://www.genomicepidemiology.org/) databases were used to find the sequence type, antibiotic resistance genes, and the plasmid types present in the isolates (4, 5). The MLST scheme of strain 1756, based on the sequences of seven housekeeping genes (*gapA2*, *infB1*, *mdh37*, *pgi1*, *phoE3*, *rpoB1*, *tonB56*), indicated that this strain belongs to a new ST type (ST2549). Antimicrobial resistance genes against β-lactams (*bla*_SHV-11_), fluoroquinolones (*oxqAB*), and fosfomycin (*fosA*) were identified on its chromosome sequence. Strain 1756 harbored a IncI1-type plasmid (pKP1756, 73,952 bp) with antimicrobial resistance genes *aph(3′)-Ia*, *aadA22*, *aac(3)-IId*, *bla*_CMY-2_, and *bla*_TEM-1B_ conferring resistance to aminoglycosides and β-lactams. IncI1 plasmids are highly prevalent in *Enterobacteriaceae* and associated with the spread of several resistance genes (6). An insertion mutation in the porin OmpK36 of strain 1756 was identified, which is likely to be associated with resistance to carbapenems in the absence of carbapenemase genes (7). This genome will facilitate exploration of the pathogenesis and antibiotic resistance of *K. pneumoniae*.

### Accession number(s).

The complete genome sequence of *K. pneumoniae* strain 1756 has been deposited in GenBank under the accession numbers CP019219 (chromosome) and CP019220 (plasmid).
